# Anti-angiotensin II type 1 receptor autoantibodies of IgG3 subclass outperform total anti-AT1R IgG1-IgG4 levels in predicting transplanted kidney antibody-mediated rejection

**DOI:** 10.3389/fimmu.2026.1776113

**Published:** 2026-03-02

**Authors:** Jakub Mizera, Karolina Marek-Bukowiec, Guido Moll, Rusan Catar, Harald Heidecke, Kai Schulze-Forster, Patryk Jerzak, Mateusz Rakowski, Karolina Władyczak, Agnieszka Hałoń, Dariusz Janczak, Piotr Donizy, Mirosław Banasik

**Affiliations:** 1 Department of Nephrology, Transplantation Medicine and Internal Diseases, Institute of Internal Diseases, Wroclaw Medical University, Wroclaw, Poland; 2 University Clinical Hospital in Wroclaw, Wroclaw, Poland; 3 Berlin Institute of Health (BIH) Center and School for Regenerative Therapies (BCRT/BSRT), Charité Universitätsmedizin Berlin, corporate member of Freie Universität Berlin, Humboldt-Universität zu Berlin, and Berlin Institute of Health (BIH), Berlin, Germany; 4 Julius Wolff Institute (JWI), Charité Universitätsmedizin Berlin, corporate member of Freie Universität Berlin, Humboldt-Universität zu Berlin, and Berlin Institute of Health (BIH), Berlin, Germany; 5 Department of Nephrology and Internal Intensive Care Medicine, Charité Universitätsmedizin Berlin, corporate member of Freie Universität Berlin, Humboldt-Universität zu Berlin, and Berlin Institute of Health (BIH), Berlin, Germany; 6 CellTrend GmbH, Luckenwalde, Germany; 7 Faculty of Health Sciences, Wroclaw Medical University, Wroclaw, Poland; 8 Department of Clinical and Experimental Pathology, Wroclaw Medical University, Wroclaw, Poland; 9 Department of Vascular, General, and Transplant Surgery, Wroclaw Medical University, Wroclaw, Poland

**Keywords:** anti-angiotensin II type 1 receptor (AT1R), anti-AT1R-directed functional/regulatory autoantibodies (AT1R-AABs/RABs), antibody-mediated rejection (AMR), G protein-coupled receptors (GPCRs), kidney transplantation (KTx), microvascular inflammation (MVI), non-HLA antibodies, Banff classification

## Abstract

**Background:**

Antibody-mediated rejection (AMR) is a leading cause of kidney allograft loss. Anti-angiotensin II type-1 receptor (AT1R) autoantibodies (AABs) have been implicated in AMR and microvascular inflammation (MVI), particularly in C4d-negative and non-HLA antibody dependent cases. Conventional assays measure only total IgG and do not assess pathogenic subclass heterogeneity. Whether IgG1-IgG4 subclass profiling improves AMR prediction has not yet been investigated.

**Methods:**

We included 143 adult kidney-transplant recipients who underwent indication biopsy between 2018 and 2025. Histopathology was classified according to Banff 2017–2022 criteria. Serum samples were analysed for total AT1R-IgG (U/mL) and AT1R IgG1–IgG4 subclasses (relative optical density). Associations with AMR were assessed using group comparisons, correlation analysis, logistic regression, ROC AUC, and quartile-based analyses.

**Results:**

AT1R-IgG3 levels were significantly elevated in AMR (Kruskal–Wallis, p = 0.0396), correlated with AMR (Spearman ρ = 0.19, p = 0.02), and demonstrated better predictive performance (AUC 0.63 vs 0.53) than total AT1R-IgG. Logistic regression showed stronger associations for IgG3 (OR 1.33, p = 0.0004) than total AT1R-IgG (OR 1.19, p = 0.029). AMR prevalence increased across IgG3 quartiles (Q1:10.5% → Q4:31.6%), while no such trend was observed for total AT1R-IgG.

**Conclusions:**

AT1R antibodies of IgG3 subclass outperform total AT1R levels in predicting AMR, revealing pathogenic antibody patterns that are not detectable through global IgG quantitation. Subclass profiling may contribute to more precise AMR risk assessment, but longitudinal and multicentre validation studies with standardized subclass-specific assays are needed to confirm these findings. Although AT1R IgG3 levels were significantly correlated with AMR, the magnitude of these associations is insufficient to support their use as an independent diagnostic marker and may only serve as a complementary AMR marker.

## 1 Introduction

Antibody-mediated rejection (AMR) remains the leading cause of kidney transplant (KTx) failure worldwide, accounting for up to 60% of graft losses beyond the first post-transplant year (1). Despite refinements in Banff Classification, a substantial proportion of AMR cases continue to be under- or misdiagnosed, contributing to graft dysfunction, long-term allograft loss, and substantial healthcare costs (2–4). This article highlights the emerging role of G-protein coupled receptor (GPCR)-directed regulatory autoantibodies (AABs/RABs) as novel biomarkers and functional mediators of the pathophysiology underlying KTx failure, in particular the need to distinguish certain immunoglobulin IgG subclasses of AABs/RABs for optimal diagnostic performance of AMR (5, 6).

It is well established in the KTx community that donor-specific anti-HLA antibodies (HLA-DSA) and C4d deposition are central immunologic indicators of AMR; however, these markers may not fully capture the complexity of alloimmune injury (4). Studies have reported that 20–50% of DSA-positive patients and 20–40% of C4d-positive recipients do not develop histologic or clinical rejection, highlighting substantial biological heterogeneity (7–9). Conversely, AMR may occur in the absence of detectable HLA-DSA or C4d, indicating the existence of additional injury pathways (3, 7). A recent large multicentre cohort analysis of 6,798 KTx recipients conducted by Sablik et al. demonstrated that microvascular inflammation (MVI), a hallmark histologic lesion of AMR, identifies high-risk phenotypes that experience worse graft outcomes even when both HLA-DSA and C4d are absent (10). These findings underscore the limitations of current diagnostic schemes and highlight the need to expand biomarker discovery beyond the traditional HLA-centric paradigm (5, 6, 11, 12).

Among the suspected alloimmune mediators, non-HLA AABs/RABs have emerged as important contributors to microvascular injury and graft failure (4–6, 11, 12). The best-studied example includes antibodies targeting GPCRs, such as the angiotensin II type-1 receptor (AT1R), known to be associated with C4d-negative AMR, endothelial dysfunction, and worse graft survival - even in DSA-negative patients (13, 14). Anti-AT1R AABs/RABs can act both agonistically, directly activating the receptor in the absence of ligand, and allosterically, enhancing the signalling response to its orthosteric ligand angiotensin II (15). Their rather recently identified pathogenic potential suggests that diagnostic algorithms should integrate both HLA and non-HLA components of the alloimmune response to enable more integrated systems biology level interpretation of the obtained results (11, 12). An important but often overlooked aspect of GPCR-directed antibodies is, that the currently established routine assays often only quantify total IgG, without considering their IgG1-IgG4 subclass composition. However, the different IgG subclasses can exhibit vastly distinct effector functions and pathogenicity (16, 17). This natural heterogeneity may possibly help explain some of the existing discrepancies and weaknesses currently reported in the literature between total anti-AT1R antibody titres and graft outcomes (17).

To date, no study has evaluated IgG subclasses of anti-AT1R antibodies in the context of AMR (11, 12). Here, we present the first subclass-specific characterisation of anti-AT1R in KTx recipients, aiming to determine whether subclass profiling improves risk stratification and correlates more accurately with AMR than total circulating anti-AT1R levels. By analysing the associations between AT1R-specific IgG subclasses and distinct patterns of renal allograft injury, we aim to advance the understanding of non-HLA-mediated alloimmune mechanisms in KTx.

## 2 Materials and methods

### 2.1 Ethics statement, study design, analysed population and inclusion/exclusion criteria

The study was conducted in accordance with the Declaration of Helsinki and it was approved by the Bioethical Committee of Wroclaw Medical University (certificate no. KB 334/2025). This study integrates both retrospective and prospective approaches. The study population included all patients who had undergone KTx and had a biopsy of the transplanted kidney between January 2018 and July 2025.

Initially, 218 records were identified, but these did not include non-HLA antibodies. At our clinic, routine assessment for anti-HLA antibodies was conducted at the time of biopsy, and if patients consent, their serum samples are stored in the clinic’s freezers after being collected for anti-HLA testing. After reviewing the availability of serum samples from the freezer, the final cohort consisted of 151 biopsy results. Thus, the retrospective analysis focused on biopsy results, while the prospective component involved measuring anti-AT1R antibodies with IgG subclasses in the stored serum samples.

In summary, the inclusion criteria were post-renal transplantation status, availability of a biopsy of the transplanted kidney performed no earlier than 2018, availability of frozen serum samples collected at the time of the biopsy, and access to the patient’s medical history along with other routinely assessed laboratory and clinical data. Patients who did not meet these criteria were excluded from the analysis.

### 2.2 Histopathological evaluation

To ensure the reliability of the histopathological assessments, only biopsy results from 2018 onwards were included, as earlier Banff classifications were considered outdated. All evaluations were performed by experienced nephropathologists (PD and AH) from the University Hospital in Wroclaw.

The assessments followed the most recent Banff classifications available at the time of each biopsy, specifically Banff 2017, Banff 2019, and Banff 2022, which were incorporated into the final analysis (3, 18, 19). Tissue samples were processed according to a standardized protocol: paraffin-embedded specimens were cut into 3-μm sections and stained with haematoxylin and eosin (H&E), periodic acid–Schiff (PAS), Jones methenamine silver (JMS), Masson’s trichrome, and immunohistochemically for C4d, ensuring consistency and reproducibility.

The evaluation focused on glomerular, interstitial, tubular, vascular, and peritubular capillary compartments. Each compartment was scored on a standardized scale from 0 (no lesions) to 3 (severe lesions), reflecting the extent of pathological changes. The mean time between transplantation and biopsy was 4.73 years, ranging from 10 days to 22.9 years.

### 2.3 AT1R total measurements and AT1R IgG1-IgG4 subclass quantitation

Blood samples were obtained as part of routine standard-of-care testing. The mean interval between biopsy and blood collection was 28 days; in 71 cases, samples were collected within ≤7 days of biopsy. Venous blood was drawn in the morning into serum separator tubes (fasting not required), centrifuged at 1500 × g for 10 min at room temperature, and serum was aliquoted and stored at −80 °C until analysis.

Total anti-AT1R IgG and anti-AT1R IgG1–IgG4 subclasses were measured by CellTrend GmbH (Luckenwalde, Germany) using ELISA kits (total IgG: #12000; IgG1: #12010; IgG2: #12020; IgG3: #12030; IgG4: #12040). Plates were pre-coated with AT1R antigen; bound antibodies were detected with subclass-specific enzyme-conjugated anti-human IgG (or anti-human IgG subclass) secondary antibodies, followed by a chromogenic substrate reaction. Total anti-AT1R IgG was reported in U/mL, whereas subclass results were reported as relative optical density (OD). All samples were assayed in duplicate and mean values were used for downstream analyses.

### 2.4 Statistical analysis

All statistical analysis was performed using Python version 3.13. The following libraries were used: pandas and numpy for data handling (20, 21), scipy.stats for standard statistical tests (22), statsmodels for regression analyses (23), and scikit-learn for ROC curve and AUC calculations (24). Data visualization was performed using matplotlib and seaborn to generate figures (25, 26).

Categorical variables were analyzed using the chi-square (χ²) test to assess associations between groups. For comparisons between two groups, either Student’s t-test (for normally distributed continuous data) or the Mann–Whitney U test (for non-normally distributed data) was applied. For comparisons across more than two groups, one-way ANOVA or the Kruskal–Wallis test was used.

Associations between continuous variables were evaluated using Pearson correlation coefficients for normally distributed data and Spearman rank correlation coefficients for non-parametric data. To assess antibody levels to predict the likelihood of antibody-mediated rejection, receiver operating characteristic (ROC) curves were constructed, and the area under the curve (AUC) was calculated.

Logistic regression models were used to quantify the association between antibody levels and the probability of AMR occurrence. Regression coefficients were transformed into odds ratios (ORs), which reflect the relative change in the odds of AMR per 1 SD increase pertaining to antibody level or optical density (OD) (standardization purposes). Statistical analyses were performed using Mann-Whitney U tests to compare AMR and non-AMR groups. Odds ratios comparing the 75th to 25th percentile (interquartile range) were calculated to provide clinically interpretable effect sizes.

Antibody levels were also categorized into quartiles to evaluate trends in AMR prevalence across different ranges of antibody concentration or OD. Differences between quartile groups were analyzed using the chi-square test.

## 3 Results

### 3.1 Population characteristics

The study comprised 151 biopsy results of 143 patients with available biobanked serum, *Banff-classified diagnostic biopsies*, and complete clinical data. Patients were divided into the following diagnostic groups: no rejection or borderline changes (N = 83), any form of T-cell–mediated rejection (TCMR; N = 35), and those with antibody-mediated rejection (AMR; N = 33). The main characteristics of the patients are summarized in **Table 1**. These cohorts were evaluated for total anti-AT1R AAB levels and IgG subclasses (IgG1–4). No relevant associations were observed for IgG1, IgG2, IgG4 (results not shown), hence only total anti-AT1R and IgG3 subclass were subjected to further analysis.

**Table 1:** Main patients’ characteristics. Red p values indicate statistically significant associations.

Assessed parameter	No rejection or borderline changes n=83	TCMR n=35	AMR n=33	P value	N missing
Time from tx to biopsy (mean) [years]	5.03; n=82	3.39; n=33	5.42; n=31	0.41	5
Recipient age at tx (mean) [years]	41.75; n=82	39.07; n=34	33.51; n=31	0.0087	4
Donor age (mean) [years]	49.25; n=65	44.69; n=26	35.76; n=25	0.001	35
CIT (mean) [min]	1066.3; n=64	949.42; n=31	1013.56; n=27	0.45	29
HLA mm total (mean)	3.41; n=56	3.58; n=26	3.68; n=22	0.66	47
C4d score	0; n=23	0; n=6	0; n=0	<0.0001	2
	1; n=37	1; n=19	1; n=0		
	2; n=15	2; n=9	2; n=13		
	3; n=6	3; n=1	3; n=20		
Recipient sex	Male; n=60	Male; n=22	Male; n=19	0.27	0
	Female; n=23	Female; n=13	Female; n=14		
No of tx before biopsy	1; n=63	1; n=29	1; n=25	0.96	7
	2; n=14	2; n=4	2; n=5		
	3; n=2	3; n=1	3; n=1		
Anti-HLA1	Positive; n=15	Positive; n=8	Positive; n=7	0.85	33
	Borderline; n=5	Borderline; n=1	Borderline; n=1		
	Negative; n=48	Negative; n=18	Negative; n=15		
Anti-HLA2	Positive; n=13	Positive; n=5	Positive; n=12	0.0094	33
	Borderline; n=6	Borderline; n=5	Borderline; n=3		
	Negative; n=49	Negative; n=17	Negative; n=8		
Anti-MICA	Positive; n=3	Positive; n=1	Positive; n=2	0.78	33
	Borderline; n=3	Borderline; n=1	Borderline; n=0		
	Negative; n=62	Negative; n=25	Negative; n=21		

The column N missing indicates how many records are missing from the total number of 151 included biopsies.

As shown in **Table 1**, C4d positivity and anti-HLA2 antibody positivity were strongly associated with AMR. To assess whether anti-AT1R AABs are independent from these factors a further analysis was performed. For each C4d score (0-3) the mean level and median value of anti-AT1R AABs (total and IgG3) was assessed. The analysis revealed no association indicating that antibody levels do not correlate with C4d positivity (**Table 2**) (p values of 0.56 and 0.22 for AT1R-IgG and IgG3 respectively).

**Table 2:** Anti-AT1R antibody levels/OD depending on C4d score.

C4d score	N	Mean AT1R IgG total level	Median AT1R IgG total level
C4d 0	N=29	10.71	10.85
C4d 1	N=56	13.38	11.67
C4d 2	N=37	12.38	9.68
C4d 3	N=27	14.56	11.25
C4d score	N	Mean AT1R IgG3 OD	Median AT1R IgG3 OD
C4d 0	N=29	0.77	0.7
C4d 1	N=56	0.86	0.76
C4d 2	N=37	0.93	0.88
C4d 3	N=27	0.89	0.89

As anti-HLA2 antibodies were strongly associated with AMR occurrence, we assessed whether mean and median anti-AT1R AAB values differ depending on anti-HLA2 status. For both, total IgG and for IgG3 subclass no associations were observed (**Table 3**) (p values 0.91 and 0.72 respectively). Considering that both, total anti-AT1R and IgG3 subclass were independent from C4d and anti-HLA2 status, indicated their potential as individual AMR diagnostic markers, which was analysed further.

**Table 3:** Anti-AT1R antibody levels/OD depending on anti-HLA2 status.

Anti-HLA2 status	N	Mean AT1R IgG total level	Median AT1R IgG total level
Borderline	14	15.52	12.68
Negative	74	12.97	11.3
Positive	30	12.89	11.02
Anti-HLA2 status	N	Mean AT1R IgG3 OD	Median AT1R IgG3 OD
Borderline	14	0.97	0.86
Negative	74	0.85	0.75
Positive	30	0.91	0.84

### 3.2 AT1R total and AT1R subclass levels in patients with no rejection, TCMR and AMR

**Figure 1:**
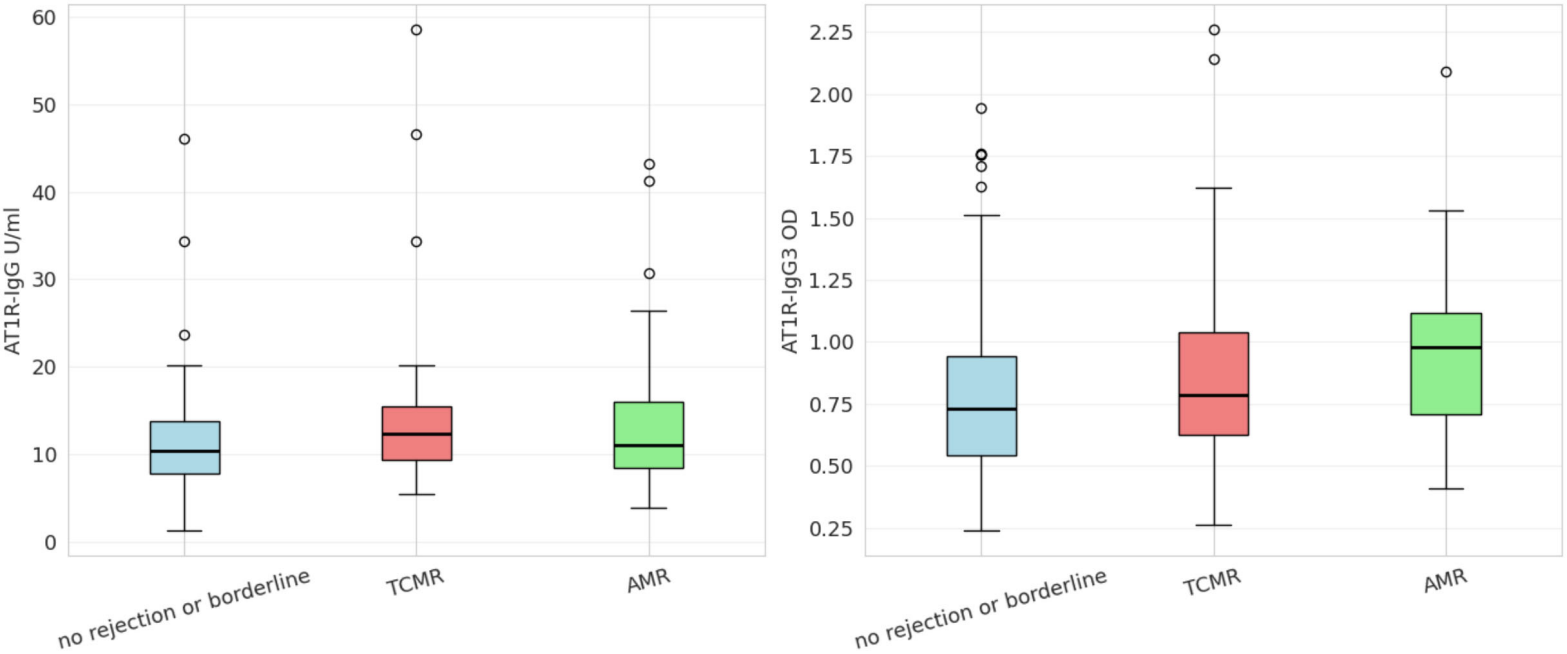
Boxplot charts illustrating AT1R total, and AT1R-IgG3 profile across histopathological diagnosis. Differences did not reach statistical significance for total antibody levels (H-statistic: 5.3289, p = 0.0696). For the IgG3 subclass, statistically significant differences were observed (H-statistic: 6.4600, p = 0.0396). The boxplots display the 95% confidence interval, with the black bold line indicating the median value. Individual dots represent outlier observations.

For total AT1R-IgG, the lowest antibody levels were observed in the no-rejection/borderline cohort (mean 11.3; median 10.37). Higher levels were detected in both the AMR and TCMR groups (mean 14.13 and 15.01; median 11.09 and 14.09, respectively) (**Figure 1**). However, none of these differences reached statistical significance. For the IgG3 subclass, the lowest mean absorbance was again observed in the no-rejection/borderline group (mean 0.81; median 0.73). Higher absorbance values were found in the TCMR (mean 0.88) and AMR (mean 0.96; median 0.98) cohorts (**Figure 1**). In this case, statistically significant differences were detected using the Kruskal–Wallis test (p = 0.0396).

### 3.3 Correlation of AT1R-IgG (total) and AT1R-IgG3 with AMR and their predictive performance

AT1R and AT1R-IgG3 were assessed for correlation with AMR diagnosis using both Pearson and Spearman coefficients. No association was observed for total AT1R (Pearson r = 0.0854, p = 0.2971; Spearman ρ = 0.0507, p = 0.5361). For the IgG3 subclass, a positive association was detected by Spearman correlation (ρ = 0.1890, p = 0.0201), whereas Pearson correlation did not confirm this association (r = 0.1320, p = 0.1063). This discrepancy suggests that the relationship between the variables may be monotonic but not linear.

**Figure 2:**
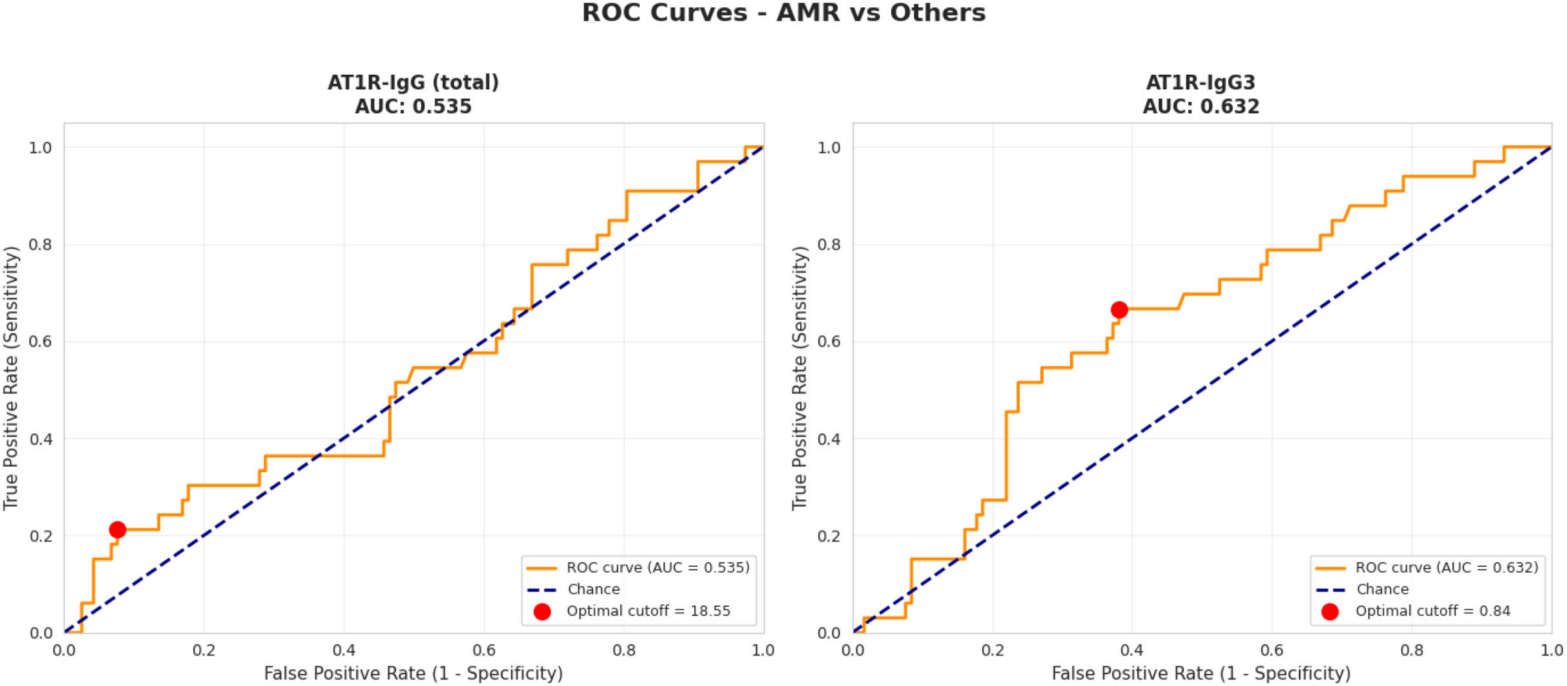
ROC AUC curves for predicting AMR based on total AT1R and AT1R-IgG3 subclass.

Anti-AT1R-IgG (total) levels and AT1R-IgG3 were evaluated for their predictive performance for AMR using ROC AUC curves. The diagnostic performance was assessed by the AUC, which measures the ability of each antibody to discriminate AMR occurrence. The AUC for total AT1R IgG was 0.535 (95% CI: 0.42–0.65), indicating no predictive ability. In contrast, IgG3 showed an AUC of 0.632 (95% CI: 0.52–0.74), suggesting limited but potentially additive predictive value (**Figure 2**).

### 3.4 Logistic regression for AMR prediction

Logistic regression analyses were performed to evaluate the association between AT1R-IgG (total) and AT1R-IgG3 levels and the risk of antibody-mediated rejection. For total AT1R-IgG, the regression coefficient was 0.178, corresponding to an odds ratio (OR) of 1.19, indicating that higher AT1R-IgG levels were modestly associated with increased risk of AMR. However, the predictive performance of the model was limited, with a cross-validated area under the receiver operating characteristic curve (AUC) of 0.53 ± 0.08.

In contrast, AT1R-IgG3 showed a stronger association with AMR. The regression coefficient was 0.287, yielding an OR of 1.33, which means that the chances for AMR occurrence rise by 33% per 1 SD increase … The predictive ability of AT1R-IgG3 was higher than total AT1R-IgG, as reflected by a cross-validated AUC of 0.63 ± 0.08, suggesting moderate discrimination between patients with and without AMR.

To better facilitate clinical interpretation, we calculated ORs comparing patients at the 75th percentile to those at the 25th percentile (IQR (interquartile range)) for each antibody. OD of AT1R-IgG3 antibodies was significantly higher in AMR patients (median 0.98 vs 0.75, p = 0.021). Comparing patients at the 75th percentile (1.01) to those at the 25th percentile (0.58), the IQR was 0,4297 with OR 1.39 (39% increase in odds). AT1R-IgG (total) antibodies showed no significant association with AMR (p = 0.536), with IQR 6,8232 and OR = 1.16.

It must be noted that the analyses were univariate and reflect rather associative than independent effects.

### 3.5 Quartile analysis

**Figure 3:**
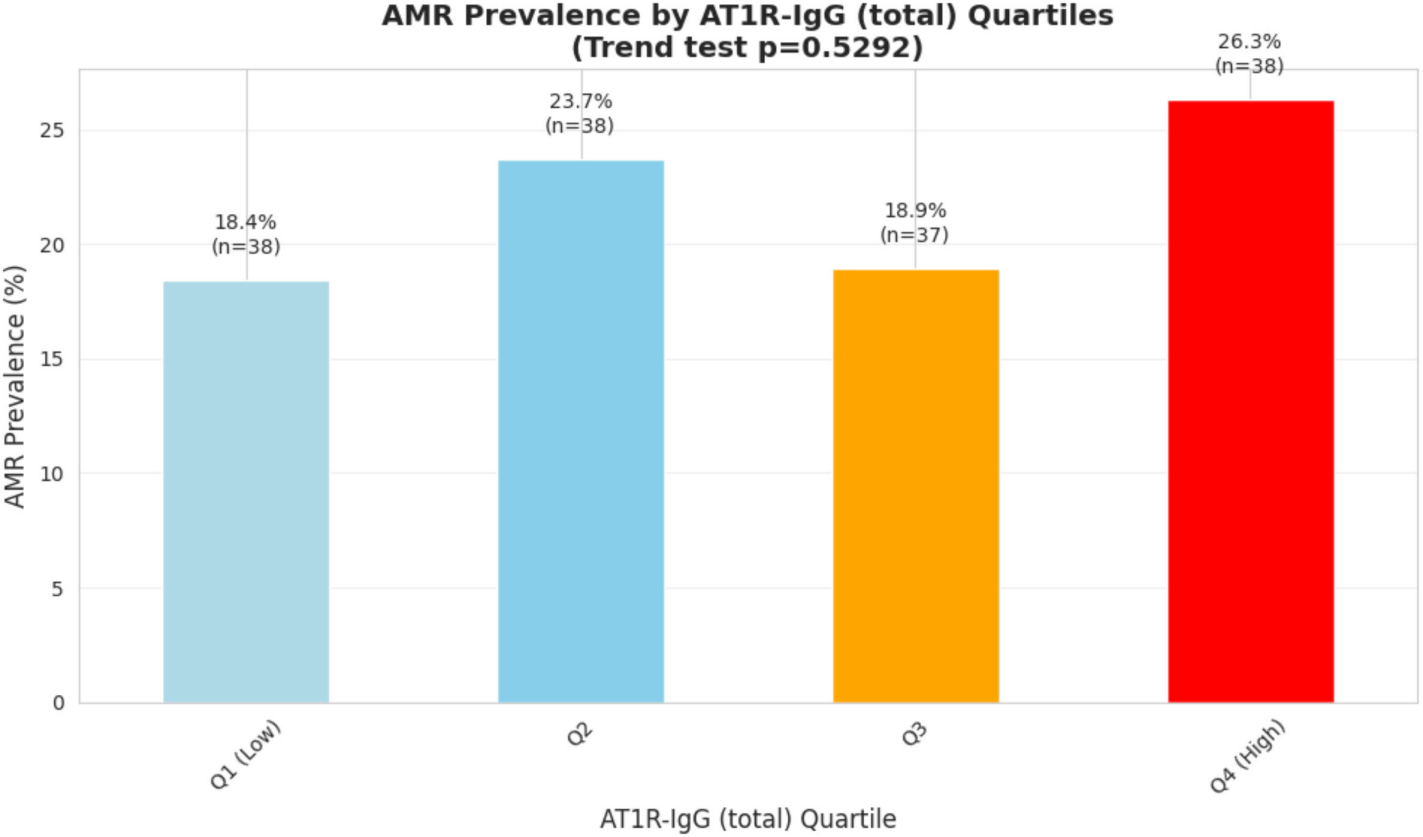
AMR prevalence in particular AT1R-IgG (total) quartiles.

We assessed the prevalence of AMR according to quartiles of total AT1R-IgG levels and AT1R-IgG3. AMR prevalence across total AT1R-IgG quartiles was not consistent, varying from 18.4% in Q1 to 26.3% in Q4, with no clear linear trend (slope = 0.019, p = 0.53, not significant) (**Figure 3**).

**Figure 4:**
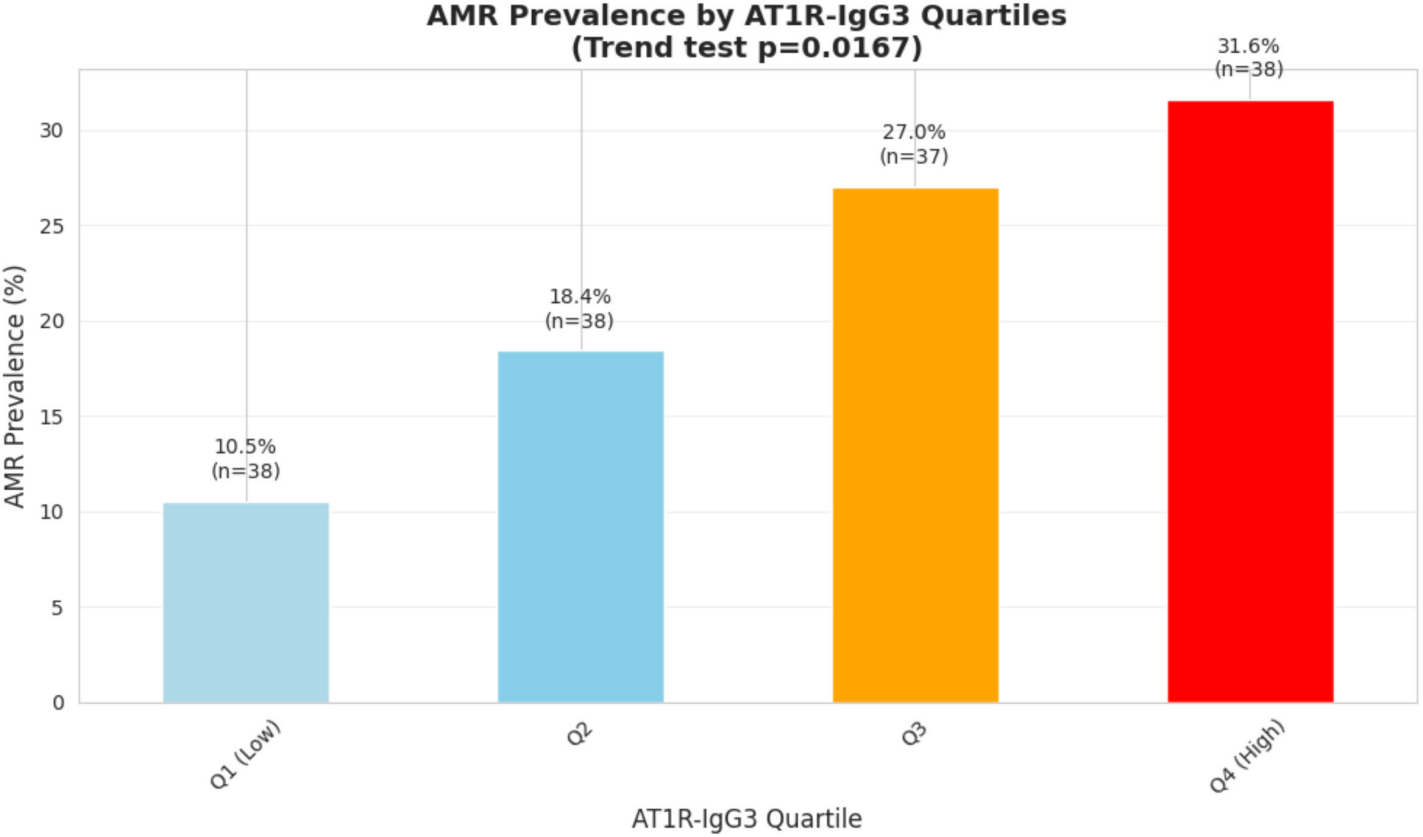
AMR prevalence in particular AT1R-IgG3 quartiles.

In contrast, the prevalence of AMR increased progressively across AT1R-IgG3 quartiles, ranging from 10.5% in the lowest quartile (Q1) to 31.6% in the highest quartile (Q4) (**Figure 4**). Trend analysis confirmed a statistically significant increase in AMR risk with higher AT1R-IgG3 levels (slope = 0.072, p = 0.017). These results suggest that AMR risk is more strongly associated with AT1R-IgG3 subclass levels than with total AT1R-IgG.

Additional comparative analyses were performed across AT1R-IgG3 quartiles to further evaluate the pattern of association with AMR. AMR prevalence was significantly higher in the upper half of the distribution (Q3–Q4: 29.3%) compared with the lower half (Q1–Q2: 14.5%), p = 0.044; OR = 2.45. The statistically significant difference between the extremes (Q1 vs Q4, p = 0.049, OR = 3.92) also confirmed that at the ends of the distribution, the discriminatory capacity is substantial. However, the lack of statistical significance in pairwise comparisons between adjacent quartiles suggests that the gradient is not strictly linear, and the differences between consecutive quartiles are relatively modest. Taken together, these results suggest that the association between AT1R-IgG3 levels and AMR exhibits characteristics of both a dose-response relationship (with increasing risk apparent from Q3 onward) and a gradual threshold effect (with the most pronounced differences at higher quartiles). The clinically relevant distinction appears to occur between patients with values below versus above the median, with those in the upper half of the distribution (Q3-Q4) demonstrating 2.45-fold increased odds of AMR compared to those in the lower half (Q1-Q2).

## 4 Discussion

In this study, we provide the first subclass-specific analysis of anti-AT1R AABs/RABs in KTx recipients and demonstrate that IgG3 anti-AT1R AABs outperform total AT1R-IgG levels in predicting antibody-mediated rejection. Indeed, the IgG3 subclass of anti-AT1R AABs outperformed total AT1R levels in all applied statistical analyses, including correlation tests, ROC AUC, logistic regression, and quartile analysis, suggesting that it may represent a significantly more reliable marker than the controversial total anti-AT1R AAB levels.

The clinical relevance of anti-AT1R AABs and their receptor has been recognized for nearly two decades, beginning with the pivotal study by Dragun et al., identifying AT1R-activating IgG antibodies in vascular rejection resistant to standard immunosuppression (13, 27). Subsequent studies confirmed an association between elevated AT1R titres, C4d-negative AMR (28), endothelial injury, and inferior graft outcomes (13, 14, 29–31). However, there are also conflicting studies indicating that non-HLA antibodies including these directed against AT1R do not contribute at all to kidney rejection episodes (32–34).

As the predictive value of total AT1R-IgG has remained inconsistent across various studies, with some cohorts reporting strong associations and others showing none, the IgG subclasses assessment emerges as a possible solution. Our findings provide a potential explanation for this variability. We speculate that only selected IgG subclasses (IgG3) exhibit true pathogenic potential.

This observation aligns with broader immunological evidence that different IgG subclasses differ in their effector and pathogenic functions. Importantly, IgG3 antibodies exhibit the strongest complement activation, highest Fcγ receptor affinity, longest hinge region, and the greatest potential for inducing endothelial activation and injury (15), thus supporting their strongest significance in our testing.

Similar patterns have also been described for HLA donor-specific antibodies (DSA), where IgG3 HLA-DSA were strongly associated with microvascular inflammation – a subtype of AMR, C4d deposition, and inferior graft survival (16).

Until now, subclass analyses in non-HLA antibodies in the transplant population have been largely absent. Our work fills this gap by demonstrating that AT1R-IgG3 follows a similar pattern to IgG3 HLA-DSA, correlating with AMR more strongly than total antibody levels. Provided evidence suggest that IgG3 AT1R antibodies may reflect a distinct, more pathogenic alloimmune phenotype.

The progressive increase in AMR prevalence across IgG3 quartiles supports its biological plausibility as marker of antibody-mediated injury. The lack of relevant findings for IgG1, IgG2, and IgG4 is notable. While IgG1 has traditionally been associated with complement activation, and IgG4 with chronic alloimmune processes, neither subclass showed correlation with AMR or Banff lesions in our cohort (35).

This may reflect true biological irrelevance, or it may be related to the semi-quantitative nature of subclass detection by OD, which lacks absolute quantification standards. Nonetheless, the clear signal in IgG3 suggests that future assay development should focus on establishing standardized quantitative subclass-specific AT1R diagnostics.

The present study also underscores the need to expand biomarker discovery beyond the HLA-centric paradigm. Sablik et al. recently demonstrated that microvascular inflammation predicts poor outcomes even in the absence of HLA-DSA and C4d (10), indicating that non-HLA mechanisms may contribute substantially to endothelial injury.

Our findings support this concept, showing that IgG3 AT1R antibodies are more often observed in AMR phenotypes regardless of total antibody titre. Thus, subclass profiling may possibly help refine diagnostic accuracy in cases where histologic AMR is present but HLA-DSA and C4d are absent.

## 5 Limitations and outlook

Several limitations of our study must be acknowledged. First, subclass detection was still based on relative OD values rather than absolute concentrations/levels; therefore, although comparative analyses across groups are valid, absolute values should be interpreted with caution.

Second, the observational, single-time-point design captures antibody levels only at the time of biopsy and does not account for the dynamic nature of AT1R antibody production. As a result, we were unable to assess temporal trajectories, fluctuations surrounding clinical events, or the evolution of IgG3 OD or total AT1R titres before transplantation or in different post-biopsy periods. This lack of longitudinal data limits causal inference and prevents distinguishing transient immunologic activation from persistent, pathogenic antibody responses.

Third, the timing of serum collection relative to biopsy was not fully synchronised across all participants, which may introduce variability in antibody measurement.

Fourth, the number of AMR cases, while clinically representative, remains moderate, underscoring the need for larger multicentre cohorts to validate these observations. Finally, mechanistic studies are required to determine whether IgG3 AT1R antibodies exert direct endothelial injury or merely reflect broader immune dysregulation.

Thus, future work should prioritise longitudinal monitoring of AT1R antibodies, ideally beginning before transplantation and continuing throughout follow-up, to elucidate antibody dynamics and their relationship to graft injury. Equally important is the development of standardised, analytically validated diagnostic assays, including subclass-specific platforms, that ensure reproducibility across laboratories.

Large, multicentre studies integrating such tools will be essential to confirm the clinical relevance of AT1R subclass profiling and its potential incorporation into routine immunologic risk assessment. More advanced, artificial intelligence driven analyses may help uncover hidden interactions of AT1R IgG providing additional insights into the role of non-HLA in AMR (36).

## 6 Conclusions

Our findings indicate that the IgG3 subclass of anti-AT1R antibodies demonstrates a stronger association with AMR than total AT1R antibody titres. Although IgG3 levels were significantly correlated with AMR, the magnitude of these associations is insufficient to support their use as an independent diagnostic marker. No significant associations were observed for IgG1, IgG2, or IgG4 subclasses. These results underscore the potential value and need for further investigation into non-HLA antibodies to define their pathophysiological relevance and potential utility in AMR risk stratification. Novel more refined and powerful analysis approaches in KTx will require well-defined, larger, prospective, multi-centric trials supported by the use of advanced non-invasive biomarker analysis and data-analysis methods, as currently established amongst others by the Paris Transplant Group and collaborators within the EU-TRAIN consortium and similar collaborative approaches (37, 38).

## Data Availability

The raw data supporting the conclusions of this article will be made available by the authors, without undue reservation.
